# Antiretroviral Drug Interactions: Overview of Interactions Involving New and Investigational Agents and the Role of Therapeutic Drug Monitoring for Management

**DOI:** 10.3390/pharmaceutics3040745

**Published:** 2011-10-21

**Authors:** R. Chris Rathbun, Michelle D. Liedtke

**Affiliations:** Department of Pharmacy: Clinical & Administrative Sciences, College of Pharmacy, University of Oklahoma Health Sciences Center, Oklahoma City, OK 73117, USA; E-Mail: Michelle-Liedtke@ouhsc.edu

**Keywords:** antiretrovirals, pharmacokinetics, drug interactions, protease inhibitors, non-nucleoside reverse transcriptase inhibitors, chemokine receptor antagonists, integrase inhibitors, therapeutic drug monitoring

## Abstract

Antiretrovirals are prone to drug-drug and drug-food interactions that can result in subtherapeutic or supratherapeutic concentrations. Interactions between antiretrovirals and medications for other diseases are common due to shared metabolism through cytochrome P450 (CYP450) and uridine diphosphate glucuronosyltransferase (UGT) enzymes and transport by membrane proteins (e.g., *p*-glycoprotein, organic anion-transporting polypeptide). The clinical significance of antiretroviral drug interactions is reviewed, with a focus on new and investigational agents. An overview of the mechanistic basis for drug interactions and the effect of individual antiretrovirals on CYP450 and UGT isoforms are provided. Interactions between antiretrovirals and medications for other co-morbidities are summarized. The role of therapeutic drug monitoring in the detection and management of antiretroviral drug interactions is also briefly discussed.

## 1. Introduction

The introduction of triple combination antiretroviral therapy has led to dramatic reductions in HIV-related morbidity and mortality [1]. Despite improvements in patient outcomes, selection of antiretroviral therapy remains challenging for clinicians due to resistance considerations, overlapping drug toxicities, and drug-drug and drug-food interactions. Drug-drug interactions among antiretrovirals are common and often require dose modification to mitigate unwanted adverse events and to sustain therapeutic concentrations. As HIV management has migrated from treatment of an acute infection to a chronic disease, the potential for antiretroviral drug interactions with medications for other chronic diseases has increased. It is important for clinicians to carefully consider the consequences of combining antiretrovirals with some drugs where the risk of adverse events or treatment failure may be increased due to unfavorable drug interactions.

This article provides an overview of common antiretroviral pharmacokinetic drug interactions, with an emphasis on newer antiretroviral agents introduced in the past five years and promising investigational agents. The mechanistic basis for drug interactions and metabolism of antiretroviral classes is briefly reviewed, followed by detailed discussion of the drug interaction potential between different antiretrovirals and with various drugs and drug classes. Recommendations for clinical management are also provided. The role of therapeutic drug monitoring in the detection and management of drug interactions is also discussed.

## 2. Mechanisms of Interaction

Drug interactions involving antiretrovirals can be classified as either pharmacokinetic or pharmacodynamic based on the mechanism of interaction. Pharmacokinetic interactions impact the absorption, distribution, metabolism, or excretion of antiretrovirals, whereas pharmacodynamic interactions result in synergistic, additive, or antagonistic drug response when they occur. Many antiretrovirals are substrates for transport proteins (e.g., organic anion-transporting polypeptide [OATP] 1B1, OATP1B3, and organic cation protein 1 [OCT1] in the liver; MDR1, breast cancer resistance protein [BCRP] and multidrug resistance protein 2 [MRP2] in the gut and liver) and can exhibit altered absorption, distribution, or excretion when coadministered with drugs that affect these proteins [2].

Interactions involving altered metabolism occur as a result of induction or inhibition of specific metabolic enzymes. Inhibition of metabolism most commonly results from competitive, non-competitive, or mechanism-based inhibition [3]. Competitive inhibition occurs when concentration of the inhibiting agent is sufficiently high to block metabolic conversion of the affected drug at the respective isoenzyme. Non-competitive inhibition occurs from allosteric inhibition, where binding to a site proximal to the catalytic binding site results in a conformational change in the catalytic site. Mechanism-based inhibition occurs when reactive intermediates bind irreversibly to the catalytic binding site [3]. Several antiretrovirals (ritonavir, amprenavir, nelfinavir, delavirdine) have been identified as mechanism-based inhibitors and are associated with clinically significant interactions with other drugs [4].

Induction of metabolism can occur when binding of drugs to nuclear receptors (e.g., pregnane X receptor [PXR], constitutive androstane receptor [CAR], hydrocarbon receptor) causes transcriptional factor activation, resulting in increased production of metabolic enzyme [5]. Individual protease inhibitors and NNRTIs have been found to be ligands for PXR and CAR, resulting in induction of specific isoenzymes or transport proteins (e.g., MDR1) that can decrease systemic exposure and increase the risk for therapeutic failure [6]. The timing of drug coadministration during pharmacokinetic interaction studies must be considered since agents that function as metabolic inducers at steady-state concentrations can exhibit inhibition during the first several weeks of therapy, leading to incorrect conclusions regarding their metabolic effects.

Pharmacogenetics can also determine whether a particular metabolic interaction is clinically significant. Polymorphic metabolism in CYP450 isoforms can influence the magnitude of change in serum concentration that is observed by either attenuating or magnifying the extent of reduction or increase. Cytochrome P450 (CYP) 3A4, CYP2B6, CYP2C9, CYPC19 and UGT all exhibit polymorphic metabolism and, therefore, can influence the significance of drug-drug interactions in specific patient populations [7–9].

## 3. Interactions between Antiretrovirals

### 3.1. Protease Inhibitors

All currently-approved HIV protease inhibitors are metabolized by CYP450, primarily CYP3A4, and are substrates for *p*-glycoprotein, making interactions likely between protease inhibitors and other antiretrovirals metabolized by CYP450. Ritonavir “boosting” is commonly employed to prolong the systemic exposure of other protease inhibitors and can further increase the potential for clinically significant interactions because of the potent inhibitive and inductive effects of ritonavir on various CYP isoforms. Ritonavir is known to autoinduce its own metabolism through CYP3A4, requiring dosage increases to maintain therapeutic concentrations when used clinically as the sole protease inhibitor (600 mg twice daily) in the antiretroviral regimen [10]. Due to the potent mechanism-based inhibitory effects of ritonavir on CYP3A4 (50% inhibitory concentration [IC_50_]: 0.05–1.4 μg/mL), ritonavir is believed to function as an inhibitor for CYP3A4 substrates with chronic administration [10,11]. In addition, the low doses of ritonavir used for pharmacokinetic boosting (100–200 mg/day) are less likely to be associated with significant induction of CYP3A4 based on the decreased extent of induction predicted by pharmacokinetic modeling at lower ritonavir doses (12% induction at 200 mg twice daily versus 45% at 500 mg twice daily) [10]. This contrasts with the apparent induction of other CYP isoforms (CYP2C9/19, CYP2B6, CYP1A2) with ritonavir [12] that can lead to clinically significant interactions with other medications [13].

Tipranavir, darunavir, saquinavir, and lopinavir all require ritonavir boosting for clinical use; therefore, the interaction profile of these agents is determined in part by the effect of ritonavir on individual isoenzymes. Many other protease inhibitors (atazanavir, indinavir, fosamprenavir) are commonly boosted with ritonavir to optimize their pharmacokinetic profile, simplify their dosing frequency, and improve their side effect profile. Interactions among protease inhibitors can be complex due to varied effects on specific CYP enzymes and may result in subtherapeutic concentrations or additive toxicity. This is exemplified by the dual interaction between lopinavir/ritonavir and fosamprenavir, where coadministration results in suboptimal serum concentrations (48% and 64% reduction in lopinavir and amprenavir systemic exposures, respectively) that cannot be reliably overcome by staggering administration or dose modification [14,15].

Alteration of the pharmacokinetics of ritonavir has also been noted when low-dose ritonavir is combined with various protease inhibitors as a boosting agent and further exemplifies the complexity of interactions between protease inhibitors. Ritonavir concentrations are lower when ritonavir is individually combined with lopinavir, fosamprenavir, and tipranavir (54%, 40%, and 90%, respectively) presumably as a result of induction of CYP3A4 (lopinavir, fosamprenavir) and/or *p*-glycoprotein (tipranavir); slightly lower ritonavir concentrations (14%) have also been reported with darunavir [16]. In contrast, increases in ritonavir systemic exposure are noted with atazanavir and indinavir (69% and 72%, respectively), potentially reflecting CYP3A4 inhibition [16]. The clinical significance of these changes varies since the ritonavir exposure required for boosting of individual protease inhibitors is different depending on the site of inhibition (gut versus gut/hepatic) [16,17] and is typically optimized prior to widespread clinical use through formal pharmacokinetic studies to achieve the desired systemic exposure for each protease inhibitor. The predicted metabolic effects of individual protease inhibitors on various CYP and UGT enzymes are depicted in Table 1.

#### Darunavir/Ritonavir

Darunavir is the newest protease inhibitor to be approved for HIV infection in the past five years and requires coadministration with ritonavir for clinical use. Ritonavir coadministration (100 mg twice daily) increased the absolute bioavailability of darunavir in healthy volunteers from 37% to 82% and produced a 14-fold increase in systemic exposure [37]. Ritonavir doses above 100 mg twice daily do not produce further increases in the area under the concentration-time curve (AUC) for darunavir [38]. Administration of darunavir/ritonavir with food increases bioavailability by 30% irrespective of whether a low or high-fat meal is administered [39].

The potential for interaction between darunavir/ritonavir and NNRTIs has been evaluated in healthy, HIV-seronegative volunteers and patients with HIV infection. Administration of darunavir oral solution (non-commercial formulation)/ritonavir (300 mg/100 mg twice daily) with efavirenz (600 mg daily) in healthy volunteers resulted in a 31% reduction in the minimum concentration (*C*_min)_ for darunavir and an increase in efavirenz AUC by 21%. Caution should be exercised with darunavir and efavirenz coadministration given the limited safety data available from clinical studies and the frequency of rash (3/12 subjects) observed during this small trial [40]. A 27% increase in nevirapine AUC and 18–47% increase in *C*_min_ were observed when nevirapine (200 mg twice daily) was administered with two separate formulations of darunavir (300 mg by oral solution, 400 mg tablet) boosted with ritonavir twice daily in HIV-infected patients. Slight elevations in darunavir AUC and *C*_min_ (9–24% and 2–23%, respectively) were also observed depending on the darunavir formulation. The observed changes are not considered to be clinically significant based on safety and efficacy results from long-term clinical studies where the combination of nevirapine and darunavir/ritonavir was used [41]. Comparable changes in nevirapine and efavirenz pharmacokinetics are projected when nevirapine and efavirenz are administered with the higher doses of darunavir/ritonavir (600 mg/100 mg twice daily) used clinically [40,41]. Etravirine 100 mg twice daily had no effect on darunavir serum concentrations; no significant interaction is predicted with higher etravirine doses (200 mg twice daily) [42].

Clinically significant interactions between darunavir/ritonavir and other protease inhibitors have also been reported. A large reduction in darunavir AUC was observed when given with lopinavir/ritonavir (400/100 mg or 533/133 mg twice daily) in HIV-infected patients. Increase in the darunavir/ritonavir dose from 600/100 mg to 1200/100 mg twice daily resulted in AUC values that remained 38% lower than darunavir/ritonavir 600/100 mg twice daily without lopinavir/ritonavir [43]. Darunavir was similarly reduced by coadministration with saquinavir/ritonavir (1000 mg/100 mg twice daily) in healthy volunteers where a 26% reduction in darunavir AUC and 42% reduction in *C*_min_ was observed [44]. Dosing strategies for darunavir to overcome the interactions with lopinavir/ritonavir and saquinavir are not established; therefore, the combination of lopinavir/ritonavir or saquinavir with darunavir is not recommended for clinical use [43,44]. No significant change in darunavir AUC was noted when darunavir/ritonavir (400/100 mg twice daily) was given with atazanavir (300 mg daily); however, atazanavir C_min_ increased by 52%. Since the safety of atazanavir systemic exposures in this range is already established, this increase is not considered clinically significant [45]. Coadministration of indinavir (800 mg twice daily) and darunavir/ritonavir (400 mg/100 mg twice daily) in healthy volunteers results in an increase in indinavir AUC and *C*_min_ (23% and 125%, respectively) and darunavir AUC and *C*_min_ (24% and 44%, respectively) compared with indinavir/ritonavir (800 mg/100 mg twice daily) alone; reduction in the indinavir dose to 600 mg twice daily is recommended if intolerance develops [46]. Raltegravir and maraviroc have no significant effect on the pharmacokinetics of darunavir/ritonavir. The effect of darunavir/ritonavir on the pharmacokinetics of raltegravir and maraviroc will be reviewed in the respective sections for these agents.

### 3.2. Non-Nucleoside Reverse-Transcriptase Inhibitors (NNRTIs)

The currently-approved NNRTIs (efavirenz, nevirapine, etravirine, delavirdine, rilpivirine) are substrates for CYP2B6 and/or CYP3A4. Rilpivirine (formerly TMC278) is the newest NNRTI to be approved in the United States and is a substrate for CYP3A4, CYP2C19, and CYP1A1, with CYP2C8/9/10 serving as alternative pathways [32]. Important food interactions exist with some NNRTIs. The bioavailability of efavirenz is increased significantly (AUC by 28%, maximum concentration [*C*_max_] by 79%) with a high-fat meal (1000 kcal, 500–600 kcal from fat) according to product labeling, resulting in a higher risk for central nervous system side effects (e.g., vivid dreams, somnolence) [47]. Coadministration with food is discouraged when initiating efavirenz but can be considered in patients who exhibit limited central nervous system effects with continued use. Etravirine exhibits improved bioavailability with food versus fasting, where systemic exposure is reduced by 51%, and should be administered with food according to manufacturer recommendations [48]. The bioavailability of rilpivirine is decreased by 43% to 50% when taken on an empty stomach or with a high-protein nutritional drink (8 grams of fat, 300 kcal) compared with a standard meal (21 grams of fat, 533 kcal) but is unaffected by a high fat meal (56 grams of fat, 928 kcal) [49].

Efavirenz and etravirine exhibit a mixed interaction profile, where induction or inhibition of specific CYP450 isoforms may be observed; whereas nevirapine primarily functions as an inducer and delavirdine as an inhibitor. Efavirenz is a weak inhibitor of CYP1A2 and CYP2D6 *in vitro*, a strong inhibitor of CYP2C9 and CYP2C19, and an inducer of CYP3A4 and CYP2B6 [6,47,50] Induction of CYP2C19 *in vivo* by efavirenz is suggested based on clinical evidence [28]. Etravirine induces CYP3A4, CYP2B6, and uridine diphosphate glucuronosyltransferase (UGT) and inhibits CYP2C9 and CYP2C19 [51]. Nevirapine induces CYP3A4, CYP2B6, CYP2C9, and possibly UGT [30,31]. Delavirdine functions primarily as an inhibitor of CYP3A4, CYP2C9, and CYP2C19 [50]. Rilpivirine exhibits weak induction of CYP3A4 and CYP2C19 at higher doses (150–300 mg) *in vivo*, weak induction of CYP1A2 and CYP2B6 *in vitro*, and is prone to having its metabolism altered by strong CYP3A4 inhibitors [52].

#### Etravirine

The drug interaction profile of etravirine with other antiretrovirals has been studied fairly extensively. Serum concentrations for some protease inhibitors are reduced by etravirine as a result of CYP3A4 induction. Increase or decrease in etravirine systemic exposure is also observed in some instances depending on the specific protease inhibitor, presumably due to altered metabolism through CYP3A4 or CYP2C9/19. Indinavir geometric mean ratios (GMRs) for AUC, *C*_max_, and *C*_min_ with and without etravirine (1600 mg twice daily; TF035 formulation) are 0.54, 0.72, and 0.24, respectively; whereas etravirine AUC, *C*_max_, and *C*_min_ are increased by approximately 50% [53]. Saquinavir AUC and *C*_max_, are 48% and 54% lower, respectively, with etravirine following a single 1200 mg saquinavir dose but are unaffected when given with low-dose ritonavir [53]. Coadministration of etravirine and atazanavir with or without ritonavir results in reduction in atazanavir *C*_min_ (38–47%) and increased etravirine systemic exposure (30–50%); the clinical significance of these changes remains to be determined [54]. Coadministration of etravirine and fosamprenavir/ritonavir (700 mg/100 mg twice daily) increased amprenavir AUC, *C*_max_, and *C*_min_, by 69%, 62%, and 77%, respectively, and should be used with caution because of the increased potential for amprenavir-related adverse events (e.g., rash, nausea) [55]. No significant interaction is noted between lopinavir/ritonavir and etravirine [53]. Darunavir/ritonavir (600 mg/100 mg twice daily) reduces etravirine AUC and *C*_min_ by 37% and 49%, respectively, but was associated with effective viral suppression in the DUET-1 and DUET-2 studies [42,56]. Full-dose ritonavir (600 mg twice daily) decreases etravirine AUC and C_max_ by 46% and 32%, respectively [57]. Etravirine serum concentrations are significantly reduced (70–80%) by tipranavir/ritonavir [58].

As a general rule, etravirine should not be administered with unboosted protease inhibitors, full-dose ritonavir, or tipranavir/ritonavir but can be safely used with darunavir/ritonavir, lopinavir/ritonavir, and saquinavir/ritonavir. Clinically significant interactions are not expected to occur between etravirine and nucleoside reverse-transcriptase inhibitors (NRTIs). Etravirine should not be coadministered with other NNRTIs because of the potential for unfavorable metabolic interactions between agents and the potential for cross resistance. Interactions between etravirine and maraviroc and raltegravir have also been reported and will be reviewed under the respective sections for chemokine receptor antagonists and integrase inhibitors.

#### Rilpivirine (TMC278)

Limited information exists on drug-drug interactions between rilpivirine and other antiretrovirals. Rilpivirine (150 mg daily) increases the *C*_max_, *C*_min_, and AUC of tenofovir (300 mg daily) by 21–24% but is not thought to be clinically significant [59]. Darunavir/ritonavir (800 mg/100 mg daily) increased rilpivirine AUC and C_min_ by 230–280% in healthy subjects; no change in darunavir or ritonavir pharmacokinetics was noted [60]. Rilpivirine pharmacokinetics are similarly increased by lopinavir/ritonavir (400 mg/100 mg twice daily) where rilpivirine AUC and *C*_min_ are modestly elevated by 52% and 74%, respectively [61]. The manufacturer states that rilpivirine dose modification is not necessary when rilpivirine is used at standards doses (25 mg daily) with either darunavir/ritonavir or lopinavir/ritonavir [62]. Coadministration of rilpivirine with other NNRTIs should be avoided due to the unfavorable interaction potential and cross-resistance concerns. Further studies are needed to ascertain the significance of interactions between rilpivirine and other antiretrovirals.

### 3.3. CCR5 Receptor Antagonists

#### Maraviroc

Maraviroc is presently the only CCR5 receptor antagonist approved for use. The bioavailability of maraviroc is reduced by 33% with a high-fat meal and by 20% with a low-fat meal; however, no food restrictions are recommended by the manufacturer [63,64]. While maraviroc demonstrates little potential to alter the metabolism of other drugs, interaction with NNRTIs and protease inhibitors is likely due to shared metabolism through CYP3A4 and altered *p*-glycoprotein function [36]. Efavirenz (600 mg daily) reduces the maraviroc AUC in healthy volunteers and HIV-infected subjects by 51% and 53%, respectively) [65,66]. Maraviroc 200 mg twice daily with efavirenz produced a maraviroc AUC comparable to maraviroc 100 mg twice daily [65]. Maraviroc systemic exposure is reduced by 53% with etravirine [67]. No significant change in maraviroc AUC from historical controls was noted when single-dose maraviroc (300 mg) was combined with nevirapine (200 mg twice daily) in HIV-infected subjects [66]. Coadministration of maraviroc (300 mg twice daily) with the protease inhibitor lopinavir/ritonavir (400 mg/100 mg twice daily) in healthy volunteers increased maraviroc AUC and *C*_max_ by approximately 4-fold and 2-fold, respectively [65]. Similar changes were observed in HIV-infected subjects [66]. Administration of maraviroc (100 mg twice daily) with saquinavir/ritonavir (1000 mg/100 mg twice daily) in healthy subjects increased maraviroc AUC and *C*_max_ by 9.8-fold and 4.8-fold, respectively [65]. Inclusion of efavirenz with either lopinavir/ritonavir or saquinavir/ritonavir yielded sustained increases in maraviroc AUC (2.5-fold and 5-fold, respectively) [65]. Maraviroc (300 mg twice daily) with either atazanavir (400 mg daily) or atazanavir/ritonavir (300 mg/100 mg daily) in healthy subjects resulted in increased maraviroc exposure (3.6-fold and 4.9-fold, respectively) [68]. In contrast, administration of maraviroc (150 mg twice daily) with tipranavir/ritonavir (500 mg/200 mg twice daily) produced no clinically significant change in maraviroc serum concentrations (GMRs of 1.02 and 0.86 for maraviroc AUC or *C*_max_, respectively) [68].

When maraviroc (150 mg twice daily) was combined with the integrase inhibitor elvitegravir (150 mg daily) and ritonavir (100 mg daily) in healthy volunteers, the GMR for maraviroc AUC was 2.86 [69]. A 37% and 28% reduction in raltegravir AUC and *C*_min_, respectively, was observed when raltegravir (400 mg twice daily) and maraviroc (300 mg twice daily) were combined in healthy volunteers, but the clinical significance of this is presently unknown due to the wide variability in raltegravir pharmacokinetics [70]. Minor reductions in maraviroc AUC and *C*_min_ (10–14%) were also observed but are not considered clinically significant.

The dose-limiting side effect for maraviroc is orthostatic hypotension; therefore, maraviroc dose reduction is necessary when combined with strong CYP450 inhibitors [63]. The manufacturer of maraviroc recommends that the dose of maraviroc be reduced to 150 mg twice daily with concomitant administration of the protease inhibitors lopinavir/ritonavir, saquinavir, atazanavir, or other strong CYP3A inhibitors [64]. This dose reduction should be employed irrespective of the inclusion of CYP3A inducers (e.g., etravirine, efavirenz) in the antiretroviral regimen. In contrast, the dose of maraviroc is recommended to be increased to 600 mg twice daily with coadministration of strong CYP3A inducers (e.g., efavirenz, etravirine) in the absence of strong CYP3A inhibitors. No adjustment in the dose of maraviroc (300 mg twice daily) is recommended when coadministered with tipranavir/ritonavir, nevirapine, raltegravir, or NRTIs [64].

### 3.4. Integrase Strand Transfer Inhibitors

Raltegravir is the only integrase inhibitor presently approved for treatment of HIV infection. Elvitegravir (GS-9137) is an investigational integrase inhibitor that is being evaluated in Phase III trials and is expected to receive approval for marketing in late 2011. Dolutegravir (S/GSK 1349572) is another investigational integrase inhibitor in earlier clinical development that demonstrates activity against raltegravir- and elvitegravir-resistant viruses [71]. Important differences exist in the interaction potential between these agents.

#### Raltegravir

Interactions between raltegravir and other antiretrovirals are less common because raltegravir is primarily metabolized by UGT1A1 [72]. Interaction between raltegravir and atazanavir with or without ritonavir has been evaluated because of the well-established inhibitory effects of atazanavir on UGT1A1 [73]. Unboosted and boosted atazanavir increase raltegravir AUC by 45–75% in healthy volunteers, but the magnitude of change is not thought to be clinically significant [74]. Similar increases in raltegravir AUC were observed with atazanavir coadministration in HIV-infected subjects [75]. The GMRs for raltegravir AUC and *C*_min_ are 0.76 and 0.46, respectively, when tipranavir/ritonavir (500 mg/200 mg twice daily) and raltegravir (400 mg twice daily) are used concurrently in healthy volunteers, but the reduction in raltegravir serum concentrations is not thought to be clinically significant due to favorable viral suppression (55% of patients [54/98] with HIV RNA <50 copies/mL) during phase III studies where the combination was used [76,77]. Etravirine decreased mean raltegravir *C*_min_ by 34% in healthy volunteers [78]. Concurrent use of raltegravir and etravirine was associated with incomplete viral suppression in a small case series (*n* = 4) of HIV-infected patients, but no clinically significant interaction was noted when etravirine and raltegravir were combined with darunavir/ritonavir [79,80]. Modest reduction (28%) in raltegravir *C*_min_ was observed with maraviroc in healthy volunteers but was not considered to be clinically significant [70]. An increase in AUC and *C*_min_ (19% and 750%, respectively) is observed when raltegravir is administered with a high-fat meal; however, raltegravir may be administered with or without food [33,81].

#### Elvitegravir (GS-9137)

Elvitegravir is metabolized by CYP3A4 and UGT1A1/UGT1A3 and requires use of a “boosting” agent to optimize its pharmacokinetic profile [82]. Bioavailability is increased approximately 3-fold when administered with food [83]. Coadministration with ritonavir or the novel investigational agent, cobicistat, prolongs systemic exposure, allowing once-daily dosing, and broadens the potential for drug-drug interactions. While the drug interaction profile of elvitegravir remains to be fully characterized, preliminary studies demonstrate no clinically significant interaction with planned co-formulated NRTIs, tenofovir disoproxil fumarate and emtricitabine, or with other NRTIs [84,85]. Similarly, no clinically relevant interaction was noted when elvitegravir was coadministered with tipranavir/ritonavir, darunavir/ritonavir, fosamprenavir/ritonavir, or etravirine [86–88]. Administration of elvitegravir (125 mg daily) with lopinavir/ritonavir (400 mg/100 mg twice daily) in HIV-infected subjects increased the elvitegravir AUC by 75%; pharmacokinetic simulations suggest that the elvitegravir dose should be decreased to 85 mg daily when used with lopinavir/ritonavir [89]. Coadministration of atazanavir/ritonavir (300 mg/100 mg daily) with elvitegravir (85 mg daily) in HIV-infected subjects produced elvitegravir systemic exposures comparable to elvitegravir/ritonavir 150 mg/100 mg daily [90]. Administration of elvitegravir (300 mg daily) and unboosted atazanavir (400 mg daily) produces similar systemic exposure to elvitegravir/ritonavir (300 mg/100 mg daily), presumably due to impaired CYP3A4 metabolism by atazanavir; however, atazanavir AUC and *C*_min_ were lower (30% and 46%, respectively) compared with historical controls [91]. Dosage reduction for maraviroc (150 mg twice daily) is recommended when used in combination with elvitegravir due to the concomitant effect of the boosting agent (*i.e.*, ritonavir, cobicistat) [69].

#### Dolutegravir (S/GSK 1349572)

Dolutegravir is a substrate for UGT1A1 and CYP3A4 (10–15%) and exhibits weak inhibitory potential for these isoenzymes based on preliminary findings [35,92]. Administration with food results in modest increases in systemic exposure depending on fat content (33% with 300 kcal, 7% fat; 41% with 600 kcal, 30% fat; and 66% with 870 kcal, 53% fat, respectively) but is not considered to be clinically significant [93]. Lopinavir/ritonavir coadministration (400 mg/100 mg twice daily) resulted in no change in dolutegravir pharmacokinetics in healthy volunteers; whereas, darunavir/ritonavir (600 mg/100 mg twice daily) decreased dolutegravir AUC and *C*_min_ by 22% and 38%, respectively [92]. Tipranavir/ritonavir (500 mg/200 mg twice daily) decreases dolutegravir AUC by 59% and *C*_min_ by 76% in healthy volunteers [94]. No dose modification for dolutegravir is considered necessary for integrase inhibitor-naïve patients when given with darunavir/ritonavir or tipranavir/ritonavir [92,94]. Administration of atazanavir (400 mg daily) or atazanavir/ritonavir (300 mg daily) with dolutegravir (30 mg daily) results in modest elevation of dolutegravir pharmacokinetics in healthy volunteers (AUC increased by 62–91%, *C*_min_ increased by 90–121%) but is considered safe [95]. Dolutegravir AUC and *C*_min_ are reduced (57% and 75%, respectively) when dolutegravir (50 mg daily) is given with efavirenz (600 mg daily) but concentrations remain 4–5-fold above the protein-adjusted IC_50_ for wild-type virus [94]. Etravirine (200 mg twice daily) significantly reduces the AUC (71%) and *C*_min_ (88%) of dolutegravir (50 mg daily) in healthy volunteers and should not be coadministered unless lopinavir/ritonavir or darunavir/ritonavir are also included [96]. No interaction is evident between dolutegravir and tenofovir disoproxil fumarate [97]. The drug-drug interaction profile of dolutegravir with maraviroc and integrase inhibitors remains to be characterized.

## 4. Antiretroviral-Non-Antiretroviral Interactions

Drug-drug interactions between antiretrovirals and medications for other co-morbidities are common and require an understanding by clinicians of which drug classes are prone to clinically-significant interactions. A comprehensive review of interactions between antiretrovirals and other medications is beyond the scope of this article; however, a summary of the interaction potential for medications widely used by clinicians for common co-morbidities is briefly discussed.

### 4.1. Acid Suppressants

Interactions between antiretrovirals and acid suppressants have been reported and occur as a result of complexation reactions, altered gastric pH, or altered CYP metabolism. Complexation reactions can occur between antacids and integrase inhibitors, resulting in decreased oral bioavailability. Integrase inhibitors function as heavy metal chelators and bind to metallic ions in the HIV integrase enzyme active site [98]. Administration of aluminum, magnesium, or calcium-containing antacids can potentially serve as binding targets for integrase inhibitors. In one clinical study, it was noted that raltegravir *C*_min_ was decreased by 67% when simultaneously coadministered with an aluminum, magnesium, and simethicone-containing antacid [99]. Coadministration of elvitegravir with an antacid led to a 41% reduction in *C*_min_ when administered simultaneously and 10–20% when separated by two hours; no interaction was evident when separated by four hours [100]. Dolutegravir AUC was reduced by 74% with simultaneous administration of an aluminum, magnesium, and simethicone-containing antacid and 26% when separated by 2 hours [101]. While the clinical significance of the serum concentration reductions in these studies is presently unknown, clinicians should advise patients to separate coadministration of antacids and integrase inhibitors by a minimum of two hours and preferably by four hours to minimize the potential for interaction. Given the potential for integrase inhibitors to bind to heavy metals, this recommendation should be applied to administration with vitamin and mineral supplements (e.g., zinc, iron). Administration of dolutegravir with a multivitamin resulted in a 33% reduction in dolutegravir AUC [101]. Impairment of absorption of other antiretrovirals can also occur with antacids. Tipranavir *C*_min_ is reduced by 29% when tipranavir/ritonavir is administered concurrently with an aluminum and magnesium-containing antacid; therefore, tipranavir/ritonavir administration should be separated from antacids by at least two hours [102].

Alteration in gastric pH can also contribute to decreased absorption of antiretrovirals. The bioavailability of atazanavir, rilpivirine, and unboosted fosamprenavir and indinavir is reduced at elevated gastric pH [103–106]. In general, chronic use of acid suppressants should be avoided in patients receiving these agents. Detailed recommendations for atazanavir use with H_2_ receptor antagonists and proton pump inhibitors in antiretroviral treatment-naïve and treatment-experienced patients are provided by the manufacturer when coadministration is necessary and are included in Table 2. Rilpivirine bioavailability was reduced by 76% when famotidine was administered two hours before rilpivirine, but no change was observed when famotidine was administered four hours after or twelve hours before rilpivirine [106]. Administration of rilpivirine with proton pump inhibitors is not recommended [107]. Fosamprenavir should be given at least two hours before H_2_ receptor antagonists or boosted with low-dose ritonavir; interactions between fosamprenavir and proton pump inhibitors do not appear to be clinically significant when administered simultaneously [103,108,109]. Indinavir systemic exposure was decreased by 34–47% when indinavir (800 mg) was administered as a single dose with omeprazole (20–40 mg) in healthy volunteer, but this effect was nullified with the addition of ritonavir (200 mg) [110]. Raltegravir AUC and *C*_max_ are increased up to 3–4-fold when administered two hours after omeprazole in healthy volunteers, presumably due to enhancement of raltegravir bioavailability at higher gastric pH [111]. Modest increases in raltegravir pharmacokinetics are also observed with omeprazole and famotidine in HIV-infected subjects but are not considered clinically significant [111,112].

Potential metabolic interactions between antiretrovirals and the proton pump inhibitors have also been reported. Tipranavir/ritonavir reduces omeprazole AUC, presumably due to induction of CYP2C19, and may require titration of the omeprazole dose to achieve desired therapeutic end points [113]. Viral rebound has been reported in 51% of patients receiving proton pump inhibitors in combination with nelfinavir for greater than 30 days [114]. Nelfinavir forms an active metabolite (M8) through CYP2C19. Omeprazole is known to be a competitive inhibitor of CYP2C19 and has been associated with a 92% reduction in the M8 metabolite [115]. Use of proton pump inhibitors and nelfinavir together should be avoided.

### 4.2. Antifungals

Metabolic interactions between azole antifungals and antiretrovirals are likely to occur due to shared metabolism through CYP3A4 and the effects of various antiretrovirals on CYP2C9/19. Protease inhibitors can increase itraconazole and ketoconazole AUC by 2–3.4-fold and may require limiting doses to 200 mg/day in some instances to minimize risk for adverse events [116–120]. Voriconazole serum concentrations are decreased (39%) when combined with low-dose ritonavir (100 mg BID); therefore, the manufacturer for voriconazole recommends that administration with boosted protease inhibitors should be avoided unless the benefit outweighs the risk [121]. Reduction in itraconazole, ketoconazole, and voriconazole systemic exposures can be expected with NNRTIs (except for delavirdine and etravirine-voriconazole) and may require azole dose adjustment to maintain therapeutic concentrations or alteration of antiretroviral therapy [29,109,122–126]. Posaconazole is less prone to interactions with antiretrovirals than older azoles because of its principal metabolism by UGT; however, lower posaconazole serum concentrations have been reported with efavirenz coadministration [127]. The pharmacokinetics of fluconazole are not significantly altered by antiretrovirals [29,128–130]. Among the echinocandins, caspofungin serum concentrations are decreased (20–40%) when caspofungin is used with strong inducers, including efavirenz and nevirapine, and may require empiric dose adjustment to maintain therapeutic concentrations [131]. Clinically significant interactions between antiretrovirals and other echinocandins (micafungin, anidulafungin) have not been described. An overview of common interactions between antifungals and antiretrovirals are summarized in Table 2.

### 4.3. Antilipidemics

HMG CoA reductase inhibitors (statins) are prone to interaction with antiretrovirals as a result of metabolism through CYP3A4 and CYP2C19 and inhibition of hepatic uptake by the OATP1B1 transporter [2,109]. Protease inhibitors can induce dyslipidemia or augment existing dyslipidemia, resulting in the need for antilipidemic therapy [132]. Statins are preferred agents for dyslipidemia because of their proven benefit on cardiovascular morbidity and mortality. Substantial increases in systemic exposure for various statins have been observed when administered with protease inhibitors. Coadministration of simvastatin (40 mg daily) with saquinavir/ritonavir (400 mg/400 mg twice daily) results in a 30-fold increase in simvastatin AUC [133]. Lovastatin is expected to exhibit significant increases in systemic exposure because of its reliance on CYP3A4-medicated metabolism [109]. A modest increase in rosuvastatin and atorvastatin AUCs is also observed when given with protease inhibitors (1.5–2-fold and 3.4–5-fold, respectively), but these agents are generally considered to be safe for use when initiated at low doses [132–136]. An exception is the use of rosuvastatin and lopinavir/ritonavir, where increases in rosuvastatin systemic exposure were associated with creatine kinase and transaminase elevations in healthy volunteers [137]. Preliminary findings with pitavastatin and several protease inhibitors (atazanavir, lopinavir/ritonavir) demonstrate no clinically significant interactions, making pitavastatin a potential alternative to atorvastatin and rosuvastatin [138,139]. Fluvastatin and pravastatin appear to be less prone to metabolic inhibition with protease inhibitors as a result of metabolism through other metabolic pathways and may also be suitable for use [132]. Titration to higher doses of statins may be necessary in individual patients receiving NNRTIs (except delavirdine) to achieve desired lipid targets because of induction of CYP3A4 [140].

### 4.4. Miscellaneous Medications

A variety of other medications commonly used in primary care settings can exhibit altered metabolism when used with antiretrovirals. In many instances, these interactions have not been formally evaluated but have been identified through case reports in the literature.

Administration of boosted PIs with levothyroxine can lead to hypothyroidism, presumably due to induction of UGT1A1 by ritonavir [141]. Atazanavir and indinavir are exceptions, where inhibition of UGT1A1 by these two agents is likely to predominate, leading to hyperthyroidism [18,142]. Chronic administration of inhaled or intranasal fluticasone with protease inhibitors is associated with development of iatrogenic Cushing syndrome [143,144]. Subsequent study has demonstrated that fluticasone serum concentrations are increased by 350-fold when given with low-dose ritonavir, likely due to impairment of CYP3A4 metabolism [145]. Elevation of other corticosteroids (e.g., budesonide, prednisolone) has also been reported with ritonavir coadministration [146,147]. Similarly, salmeterol serum concentrations can be increased by boosted and unboosted protease inhibitors, increasing the risk for cardiac arrhythmias, and should not be coadministered [148]. Warfarin metabolism can be altered by a variety of antiretrovirals due to inhibition or induction of CYP2C9; a summary of specific interactions between antiretrovirals and warfarin is included in Table 2 [13]. The erectile dysfunction agents (sildenafil, taladafil, vardenafil) all exhibit significant potential for interaction with antiretrovirals metabolized by CYP3A4 and require dosage adjustment when beginning therapy to minimize the risk for symptomatic hypotension and priapism [109]. The interaction potential and clinical management of antiretrovirals with anticonvulsants and antimycobacterials are reviewed in Table 2.

## 5. Therapeutic Drug Monitoring to Manage Drug-Drug Interactions

### 5.1. Rationale for Therapeutic Drug Monitoring

Therapeutic drug monitoring (TDM) is the practice of dosing medications in response to plasma drug concentrations with the goal of maintaining concentrations within a clinically determined therapeutic target range. The aim is to optimize the clinical efficacy of a medication while minimizing or eliminating its concentration-dependent toxicities. In order for TDM to be valuable, a relationship between plasma concentrations and efficacy and/or toxicity must be established with a therapeutic agent. The role of TDM in regard to antiretrovirals continues to be largely undefined. Various small prospective and retrospective studies have displayed benefits in achieving virologic outcomes and minimizing toxicity when TDM is used in routine practice [157–159]. However, experts agree that large, well-designed trials are still needed to clearly delineate the role of TDM and to identify which patients may benefit most from its use [160,161]. One of the proposed populations where TDM may be advantageous is for patients at risk for clinically significant drug interactions [160]. Given that patients receiving antiretrovirals may experience complex, often unpredictable interactions with concomitant medications, the use of TDM can potentially allow examination and correction of altered serum concentrations prior to the development of virologic failure or adverse events.

The rationale for TDM in patients treated with antiretrovirals is primarily based on the large inter-patient variation in serum concentration that some agents exhibit when the same dose is administered to different patients [157,162,163]. This variation may be due to many factors including alteration in absorption, membrane transport, hepatic metabolism, and drug interactions. It follows that, if plasma drug concentrations can be reliably maintained within established ranges for efficacy and safety, clinicians can improve treatment response and limit toxicity risks for individual patients. Antiretroviral resistance development is associated with subtherapeutic drug levels and intermittent antiretroviral administration [164]. If clinicians are able to determine which patients are at risk for resistance development earlier in treatment (e.g., suboptimal drug concentrations due to erratic adherence from poor drug tolerability), dose alteration could potentially ameliorate drug intolerance and avoid the acquisition of antiretroviral resistance altogether. Another theoretical benefit of TDM is optimization of antiretroviral regimens in patients who have limited treatment options secondary to either extensive resistance or economic constraints. Adjusting doses to increase virologic suppression and limit toxicity might allow providers to use individual antiretrovirals longer without needing to change therapy.

The role of TDM for different antiretroviral classes varies. Protease inhibitors and NNRTIs display greater than 100-fold variation in pharmacokinetic parameters (AUC, *C*_max_, *C*_min_) associated with efficacy and toxicity end points [157,162,163,165]. This characteristic, combined with data supporting concentration-effect and concentration-toxicity relationships, makes protease inhibitors and NNRTIs the most likely candidates for TDM [157,161,166]. The CCR5 receptor antagonist, maraviroc, is a substrate for CYP3A4 and is highly susceptible to serum concentration changes when coadministered with strong CYP3A4 inducers or inhibitors. However, limited data exist on an observed association between maraviroc trough concentrations and virologic response to support it as a candidate for TDM [64,109,167]. The fusion inhibitor, enfuvirtide, and the integrase inhibitor, raltegravir, have relatively few drug interactions with other antiretrovirals or medications and do not utilize the CYP450 system for metabolism; therefore, no compelling role for TDM exists for them [165]. The clinical effect of NRTIs is related to the intracellular concentration of their active triphosphate; therefore, plasma concentrations are less predictive of clinical response or toxicity [168]. These agents also are predominantly eliminated renally and, with the exception of tenofovir, exhibit minimal potential for metabolic interactions, making plasma concentration monitoring of limited utility for NRTIs in general.

### 5.2. Barriers to Therapeutic Drug Monitoring

Barriers to routine application of TDM in clinical practice remain fairly formidable. Significant debate continues about which pharmacokinetic parameter(s) provides the best measure for drug efficacy or toxicity. Researchers have used a combination of *C*_min_, *C*_max_, AUC, and concentration ratio to assess efficacy and toxicity outcomes [169]. At the present time, the C_min_ provides the best measure of virologic effect for most clinical scenarios without requiring complex calculations and population parameters [165]. However, definitive target values remain to be defined. Consensus guidelines have identified target trough concentrations for TDM in antiretroviral-naïve patients with wild-type virus; these values are listed in Table 3 [109,160,165,170]. It is not clear if individual targets are applicable to both antiretroviral-naïve and antiretroviral-experienced patients. In the case of antiretroviral-experienced patients, resistance mutations may increase the target antiretroviral serum concentration required to achieve viral suppression. Several studies have attempted to define the antiretroviral inhibitory quotient, which represents a combination of both pharmacokinetic/pharmacodynamic properties of the medication as well as virus-specific resistance information from either genotypic or phenotypic testing. Several different IQs have been proposed, with the PIQ (phenotype-based) and GIQ (genotype-based) discussed most predominately. The PIQ represents the ratio between the *C*_min_ and the IC_50_ or IC_90_ measured by phenotypic resistance testing. The GIQ is defined as the ratio between *C*_min_ and the number of resistance-associated mutations [161,165,169]. At the present time, no agreement exists on which inhibitory quotient is most closely tied to positive therapeutic outcomes. Preliminary target cutoffs have been proposed but require further study to correlate antiretroviral dose with viral response in patients with underlying resistance. It may be possible to overcome low- to mid-level resistance by utilizing IQ targets to individualize antiretroviral doses, giving clinicians an effective tool to achieve virologic success in highly treatment-experienced patients with numerous resistance mutations [161]. It is possible that targets will also vary based on the extent of synergy in individual antiretroviral combinations. Additionally, most protease inhibitors and NNRTIs are highly bound to plasma proteins; however, current targets are expressed as total plasma concentrations and do not correct for altered protein binding.

Laboratory validity is also a potential barrier to the clinical application of TDM results in different countries. Various methodologies can be employed by different clinical laboratories to measure antiretroviral plasma concentrations [160,171]. There is currently no standardized procedure or regulated quality control for determination of antiretroviral serum concentrations in laboratories in most commercial settings, making it difficult to apply specific laboratory results to proposed serum concentration targets established in research studies. Within the United States, the availability of reputable laboratories to perform antiretroviral serum concentration measurements is also limited, necessitating transport of patient samples that can extend turnaround time and decrease the clinical utility of laboratory results for patient management.

### 5.3. Role of Therapeutic Drug Monitoring for Drug-Drug Interactions

Despite the barriers that exist to widespread acceptance, clinical application of TDM has increased in the past few years, particularly in some European countries. In the United States, TDM continues to be primarily used in a research capacity. Treatment guidelines in several countries offer some limited information and guidance in the use of TDM [109,172–174]. There is uniform agreement between guidelines that routine application of TDM in patients treated with antiretrovirals is currently not recommended based on clinical evidence [109,169,172–174]. Most guidelines, including the United States Department of Health and Human Services Antiretroviral Treatment Guidelines [109], state that TDM may be useful in specific patient populations that could be at risk for subtherapeutic or supratherapeutic antiretroviral concentrations. One of the populations identified is patients with potential drug-drug interactions [109,161,169,172–174]. Two recent studies have investigated which patients may be at greater risk for experiencing drug-drug interactions. The factors identified include older patients (>42 years old), presence of more than three co-morbid conditions, treatment with more than five non-antiretroviral agents, antiretroviral therapy consisting of more than three antiretroviral medications, and use of either a protease inhibitor- or NNRTI-containing regimen [175,176]. In patient populations such as these that may be at increased risk for clinically significant drug-drug interactions, TDM may be prudent at the initiation of therapy or with medication changes to assure successful attainment of treatment outcomes. In addition, TDM would be advised for any patients with a high potential for drug-drug interactions that are experiencing unexpected clinical outcomes (e.g., sluggish viral load response, excessive adverse events). Therapeutic drug monitoring may provide insight into the cumulative effect on individual drug concentrations for antiretroviral regimens that involve multiple drug-drug interactions and whether antiretroviral doses are appropriate to achieve desired therapeutic end points. Practitioners who wish to perform TDM should keep in mind the limitations discussed and utilize guideline targets if possible. Expert consultation is advised for management of complex cases where extensive antiretroviral drug resistance is involved.

## 6. Conclusions

Combination antiretroviral therapy is highly effective in reducing HIV-related morbidity and mortality; however, clinicians must balance treatment outcomes with the potential for complex drug-drug interactions between antiretroviral and with medications for other chronic diseases. Antiretrovirals are prone to drug-drug interactions as a result of shared metabolism through CYP450 and UGT isoforms and binding to membrane transporters. Protease inhibitors and NNRTIs, including the newer agents darunavir and etravirine, commonly alter the metabolism of other medications. The clinical significance of drug interactions between rilpivirine, the newest NNRTI, and other antiretrovirals remains to be determined. Maraviroc is the only CCR5 receptor antagonist presently available and frequently requires dose modification with other antiretrovirals. The integrase inhibitor, raltegravir, and investigational agent, dolutegravir, are less prone to clinically significant drug interactions; whereas the investigational agent, elvitegravir, is more likely to exhibit interaction with other drugs because of coadministration with a pharmacokinetic enhancer (e.g., ritonavir, cobicistat). Important drug interactions exist between antiretrovirals and acid suppressants, antilipidemics, and medications for other chronic diseases. Therapeutic drug monitoring may represent a viable mechanism to identify and manage drug interactions in individual patients, but important barriers exist to widespread application in clinical practice.

## Figures and Tables

**Table 1.** Metabolic Effects for Antiretrovirals [6,12,18–36].

		**Predicted Enzyme Effect**						
	**Antiretroviral**	**3A4**	**2B6**	**2C9**	**2C19**	**2D6**	**1A2**	**UGT**
**PIs**	Atazanavir		–		–a	–		
	Darunavir/r					–a		
	Fosamprenavir			–	–a	–	–	–
	Indinavir		–	–	–	–a	–	
	Lopinavir/r					–a		
	Nelfinavir		–			–		
	Ritonavir	b						
	Saquinavir		–	–a	–	–	–	–
	Tipranavir/r							
**NNRTIs**	Delavirdine		–			–a	–	–
	Efavirenz							
	Etravirine					–	–	
	Nevirapine				–	–	–	
	Rilpivirine			*		*		*
**INSTI**	Raltegravir	–	–	–	–	–	–	
	Elvitegravir/r		*	*	*	*	*	
	Dolutegravir		*	*	*	*	*	
**CRA**	Maraviroc		–	–	–	–	–	–
							

The predicted metabolic effects of antiretroviral agents on various cytochrome (CYP) P450 isoenzymes and uridine diphosphate glucuronosyltransferase (UGT) are illustrated according to the following: 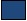 inhibition, 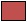 induction, 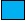 mixed induction/inhibition, 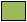 substrate, [□] no significant effect, [*] not determined. The clinical significance of specific interactions between antiretrovirals and other drugs will be determined by the therapeutic and toxicity indices of the affected drug(s). The use of low-dose ritonavir for pharmacokinetic boosting is denoted by lowercase “/r” following individual antiretrovirals.

a Enzyme not affected at clinically relevant antiretroviral concentrations.

b Autoinduction of CYP3A4 by ritonavir is observed during the first 2 weeks of therapy, but CYP3A4 inhibition is most commonly evident with chronic therapy. PIs = protease inhibitors, NNRTIs = non-nucleoside reverse-transcriptase inhibitors, INSTIs = integrase strand transfer inhibitors, CRA = CCR5 receptor antagonist

**Table 2.** Select antiretroviral drug interactions with medications for other comorbidities.

**Medication**	**Antiretroviral**	**Predicted Effect**	**Management**

**Acid-Suppressants** [106,107,109,110,115,149]			

Antacids	Integrase Inhibitors	⇩RAL, ETG, DTG	Consider separating administration by 4 hours. Take ATV 2 hours before or 1 hour after antacids Take FPV simultaneously or 2 hours before or 1 hour after antacids Take TPV 2 hours before or 1 hour after antacids
	Atazanavir ± ritonavir	⇩ATV	
	Fosamprenavir (unboosted)	⇩APV	
	Tipranavir/ritonavir	⇩TPV	

H_2_-Receptor Antagonists (H2A)	Atazanavir/ritonavir	⇩ATV	Administer boosted ATV simultaneously or ≥ 10 hours after H2A; do not exceed 40 mg famotidine dose equivalent BID (ART-naïve) or 20 mg dose equivalent BID (ART-experienced). For TDF-containing ART (ART-experienced), use ATV 400 mg + RTV 100 mg. Administer unboosted ATV 2 hours before or ≥ 10 hours after H2A; do not exceed dose equivalent of famotidine 20 mg as a single dose or 20 mg BID total daily dose (ART-naïve). Avoid coadministration in ART-experienced patients. Take FPV ≥ 2 hours before H2A; consider ritonavir boosting. Administer H2A either 12 hours before or 4 hours after RPV.
	Atazanavir (unboosted)	⇩ATV	
	Fosamprenavir (unboosted)	⇩APV	
	Rilpivirine	⇩RPV	

Proton Pump Inhibitors (PPIs)	Atazanavir ± ritonavir	⇩ATV	PPIs not recommended with unboosted ATV or in ART-experienced patients. Do not exceed omeprazole 20 mg dose equivalent; separate dosing by ≥ 12 hours (ART-naïve) Avoid coadministration; consider ritonavir boosting Avoid coadministration (decreased active metabolite formation) Monitor for SQV-related adverse effects Consider omeprazole dose increase. Do not coadminister with proton pump inhibitors
	Indinavir (unboosted)	⇩IDV	
	Nelfinavir	⇩active metabolite (M8)	
	Saquinavir/ritonavir	⇧SQV	
	Tipranavir/ritonavir	⇩Omeprazole	
	Rilpivirine	⇩RPV	

**Anticoagulants** [13,109]			

Clopidogrel	Etravirine	⇩clopidogrel active metabolite	Avoid coadministration (potential for decreased clopidogrel active metabolite formation)

Warfarin	Boosted PIs, Nelfinavir	⇩Warfarin	Adjust warfarin dose accordingly based on INR. Adjust warfarin dose accordingly based on INR. Adjust warfarin dose accordingly based on INR. Adjust warfarin dose accordingly based on INR.
	Unboosted PIs (except NFV)	⇧/⇩Warfarin	
	Efavirenz, Etravirine, Delavirdine	⇧Warfarin	
	Nevirapine	⇩Warfarin	

**Anticonvulsants** [109,150]			

Carbamazepine (CBZ)	Boosted PIs (except DRV)	⇩PI, ⇧CBZ	Monitor carbamazepine serum concentrations and HIV viral load; **do not** coadminister with LPV/r once-daily. Monitor carbamazepine serum concentrations Monitor carbamazepine serum concentrations and HIV viral load; consider ritonavir boosting Monitor carbamazepine serum concentrations and HIV viral load
	Darunavir/ritonavir	⇧CBZ	
	Atazanavir, Fosamprenavir (unboosted)	⇩PI	
	Efavirenz	⇩EFV, ⇩CBZ	

Lamotrigine	Boosted PIs	⇩LMG	Monitor lamotrigine serum concentrations

Phenytoin (PHT)	Boosted PIs	⇩PHT, ⇩PI (⇧APV)	Monitor phenytoin serum concentrations and HIV viral load; **do not** coadminister with LPV/r once-daily. Monitor phenytoin serum concentrations and HIV viral load; consider ritonavir boosting Monitor phenytoin serum concentrations and HIV viral load. Increase MVC dose to 600 mg BID.
	Atazanavir, Fosamprenavir (unboosted)	⇩ATV, APV	
	Efavirenz, Etravirine	(⇧PHT)	
	Maraviroc	⇩MVC	

Valproic Acid (VPA)	Lopinavir/ritonavir	⇩VPA, ⇧LPV	Monitor valproic acid serum concentrations and response; monitor for LPV toxicity Monitor for ZDV-related adverse events
	Zidovudine	⇧ZDV	

**Corticosteroids** [109,151]			

Dexamethasone	PIs	⇩PIs	Use with caution; consider alternative corticosteroid

Budesonide	Boosted PIs	⇧Budesonide	Avoid chronic coadministration unless potential benefit outweighs risk for systemic corticosteroid adverse effects

Fluticasone (inhaled/intranasal)	PIs, Delavirdine	⇧Fluticasone	Avoid chronic coadministration unless potential benefit outweighs risk for systemic corticosteroid adverse effects

Prednisone	Boosted PIs	⇧Prednisolone	Avoid chronic coadministration unless potential benefit outweighs risk for systemic corticosteroid adverse effects

Triamcinolone	Boosted PIs	⇧Prednisolone	Avoid chronic coadministration unless potential benefit outweighs risk for systemic corticosteroid adverse effects

**Antifungals** [68, 109,113,123–126,131,152]			

Fluconazole	Tipranavir/ritonavir	⇧TPV/r	Fluconazole doses >200 mg/d not recommended; consider alternative PI or another antiretroviral class Monitor for ETV- and NVP-related adverse events
	Etravirine, Nevirapine	⇧ETV, NVP	

Itraconazole	PIs	(⇧ITZ, ⇧PI)	Consider monitoring itraconazole serum concentrations or not exceeding itraconazole 200 mg/day Monitor itraconazole serum concentrations and antifungal response Decrease MVC dose to 150 mg BID.
	NNRTIs (except DLV)	⇩ITZ, ⇧ETV, NVP	
	Maraviroc	(⇧MVC)	

Posaconazole	Atazanavir ± ritonavir	⇧ATV	Monitor for ATV-related adverse events Consider alternative antifungal or monitoring posaconazole serum concentrations
	Efavirenz	⇩PCZ	

Voriconazole	Boosted PIs	⇩VCZ	Avoid coadministrationr with boosted PIs; consider monitoring voriconazole serum concentrations Increase voriconazole maintenance dose to 400 mg BID and decrease EFV by 50% Monitor for NNRTI-related adverse effects, antifungal response and/or voriconazole serum concentrations
	Efavirenz	⇩VCZ, ⇧EFV	
	Nevirapine, Rilpivirine	(⇩VCZ, ⇧NVP, RPV)	

Ketoconazole	PIs	⇧KTZ	Monitor for ketoconazole-related adverse effects; consider not exceeding ketoconazole 200 mg/day Avoid coadministration; monitor antifungal response and/or ketoconazole serum concentrations Monitor for antifungal response and ETV- and RPV-related adverse events. Decrease MVC dose to 150 mg BID.
	Efavirenz, Nevirapine	⇩KTZ, ⇧NVP	
	Etravirine, Rilpivirine	⇩KTZ, ⇧ETV, RPV	
	Maraviroc	⇧MVC	

Caspofungin	Efavirenz, Nevirapine	⇧Caspofungin	Consider increasing caspofungin dose to 70 mg.

**Antilipidemics** [109,137–139]			

Statins (Simvastatin, Lovastatin)	PIs	⇧Statin	Do not coadminister due to increased risk for serious adverse events; consider atorvastatin, rosuvastatin, pitavastatin, fluvastatin, pravastatin as alternatives beginning at low doses Titration of statin dose may be necessary to achieve desired treatment response.
	Efavirenz, Etravirine, Nevirapine	⇩Statin	

Rosuvastatin	Lopinavir/ritonavir	⇧Rosuvastatin	Use with caution (increased risk for serious adverse events)

**Antimycobacterials** [109,153–156]			

Clarithromycin	Atazanavir ± ritonavir	⇧Clarithromycin	Reduce clarithromycin dose by 50% due to risk for QTc prolongation Monitor for clarithromycin-related adverse effects; reduce clarithromycin dose by 50% for patients with CrCl 30–60 mL/min and by 75% with CrCl <30 mL/min Consider alternatives to clarithromycin Decrease MVC dose to 150 mg BID
	Boosted PIs (except ATV)	⇧Clarithromycin	
	NNRTIs (except DLV)	⇩Clarithromycin	
	Maraviroc	⇧MVC	

Rifampin	PIs	⇩PIs	Do not coadminister; increased risk for hepatotoxicity Monitor virologic response; consider increasing EFV dose to 800 mg QD for patients >60 kg Do not coadminister Increase RAL dose to 800 mg BID Do not coadminister
	Efavirenz	⇩EFV	
	NNRTIs (except EFV)	⇩NNRTIs	
	Raltegravir	⇩RAL	
	Maraviroc	⇩MVC	

Rifabutin	Boosted PIs, Atazanavir (unboosted)	⇧Rifabutin	Decrease rifabutin to 150 mg QOD or 3×/week; some experts recommend 150 mg QD or 300 mg 3×/week due to increased risk of treatment failure with 150 mg 3×/week dosing. TDM for rifabutin recommended. Decrease rifabutin to 150 mg QD or 300 mg 3×/week; increase IDV to 1000 mg q8 hours Increase rifabutin to 450 mg daily Do not use rifabutin when ETV is combined with boosted PIs Do not coadminister
	Fosamprenavir, Indinavir (unboosted)	⇧Rifabutin	
	Efavirenz	⇩Rifabutin	
	Etravirine	⇩Rifabutin, ⇩ETV	
	Rilpivirine, Delavirdine	⇩RPV, DLV	

**Erectile Dysfunction Agents** [109]			

PDE5 Inhibitors (Sildenafil, Tadalafil, Vardenafil)	PIs, Delavirdine	⇧PDE5 Inhibitor	Begin with sildenafil 25 mg Q48 hours. Begin with tadalafil 5 mg; do not exceed 10 mg Q72 hours. Begin with vardenafil 2.5 mg; do not exceed 2.5 mg Q72 hours Increase in PDE5 inhibitor dose may be necessary
	Etravirine	⇩PDE5 Inhibitor	

**Miscellaneous** [18,109,141,142]			

Levothyroxine	Boosted PIs (except ATV, IDV)	⇩Levothyroxine	Monitor TSH and titrate levothyroxine dose accordingly Monitor TSH and titrate levothyroxine dose accordingly
	Atazanavir, Indinavir	⇧Levothyroxine	

Salmeterol	PIs ± ritonavir	⇧Salmeterol	Do not coadminister due to increased risk for arrhythmias

APV = amprenavir, ART = antiretroviral therapy, ATV = atazanavir, CBZ = carbamazepine, DLV = delavirdine, DRV = darunavir, DTG = dolutegravir, FPV = fosamprenavir, EFV = efavirenz, ETG = elvitegravir, ETV = etravirine, FLU = fluconazole, IDV = indinavir, ITZ = itraconazole, KTZ = ketoconazole, LMG = lamotrigine, LPV = lopinavir, MVC = maraviroc, NNRTIs = non-nucleoside reverse-transcriptase inhibitors, NVP = nevirapine, PCZ = posaconazole, PDE5 = phosphodiesterase-5 inhibitors, PHT = phenytoin, PIs = protease inhibitors, RAL = raltegravir, RPV = rilpivirine, TPV = tipranavir, TSH = thyroid stimulating hormone, VCZ = voriconazole.

**Table 3.** Minimum antiretroviral trough concentration targets for treatment-naïve and treatment-experienced patients [109].

**Antiretroviral Drug**	**Minimum Trough Concentrations (Treatment-Naïve Patients) [ng/mL]**	**Minimum Trough Concentrations (Treatment-Experienced Patients) [ng/mL]**
Atazanavir	150	
Darunavir^a^		3,300
Fosamprenavir	400	
Indinavir	100	
Lopinavir/ritonavir	1,000	
Nelfinavir^b^	800	
Ritonavir		
Saquinavir	100–250	
Tipranavir		20,500
Efavirenz	1,000	
Nevirapine	3,000	
Etravirine^a^		275
Maraviroc		>50
Raltegravir^a^		72

a Target serum concentrations represent median trough concentrations from clinical trials;

b Target serum concentrations represent the active metabolite (M8)
